# Anion-Doped Perovskite Oxygen-Permeable Membranes Fabricated via an Improved One-Step Thermal Processing Approach

**DOI:** 10.3390/ma17235929

**Published:** 2024-12-04

**Authors:** Yongqiang Niu, Wanglin Zhou, Shuyang Ni, Zhengkun Liu, Guangru Zhang, Wanqin Jin

**Affiliations:** 1 State Key Laboratory of Materials-Oriented Chemical Engineering, College of Chemical Engineering, Nanjing Tech University, 30 Puzhu Road(S), Nanjing 211816, China; yongqiang@njtech.edu.cn (Y.N.); wlzhou@njtech.edu.cn (W.Z.); nisy0418@163.com (S.N.); wqjin@njtech.edu.cn (W.J.); 2 Quzhou Membrane Material Innovation Institute, Nanjing Tech University, Quzhou 324000, China

**Keywords:** anion-doped perovskite, fluorine, elemental dissolution, oxygen-permeable membrane, hollow fiber membrane

## Abstract

Anion-doped perovskite membranes with a hollow fiber geometry have excellent oxygen separation performance. However, during the fabrication process of hollow fiber membranes, soaking the precursor in deionized water leads to elemental dissolution, especially anion dissolution. To prevent metal and anion element dissolution, an improved one-step thermal processing approach was proposed in which saturated solutions were used as internal and external coagulation baths, effectively controlling the stoichiometric ratio. Compared with using deionized water as internal and external coagulation baths, using a fluorine-containing saturated solution increased the oxygen flux of the membrane by 21% at 900 °C. The oxygen permeability of the fluorine-doped oxide membrane reached 6 mL cm^−2^ min^−1^ at 900 °C, with an oxygen flux exceeding 1 mL cm^−2^ min^−1^ at 700 °C, meeting commercial oxygen separation membrane standards. Anion doping and stability enhancement strategies could further advance the development and practical use of oxygen separation membranes.

## 1. Introduction

Dense perovskite membranes theoretically offer 100% oxygen selectivity, as oxygen is transported in an ionic form rather than as molecules [1]. Over the past several decades, significant advancements have been made in the development of oxygen separation membranes, particularly in terms of material innovation [2]. Perovskite membranes have garnered significant attention in recent years due to their desirable characteristics, including oxygen permeability and easy preparation [3]. These characteristics make them promising for various applications, such as pure oxygen production [4], oxygen-enriched combustion [5], solid oxide fuel cells [6], and membrane-catalyzed oxidation [7]. Unsatisfactory oxygen permeability is the main issue hindering the large-scale practical application of perovskite membranes at low temperatures [8]. Therefore, there is an urgent need to develop oxygen-permeable membranes with superior oxygen permeability at 500–800 °C to accelerate the commercial application of perovskite membranes.

Addressing these issues from the perspective of material innovation remains the primary focus of research. Ba_0.5_Sr_0.5_Co_0.8_Fe_0.2_O_3−δ_ (BSCF) perovskite oxide is regarded as one of the most promising membrane materials for industrial applications [9]. However, the low oxygen permeability and poor stability of BSCF materials at 500–800 °C hinder their commercial application [10]. Cations are usually doped or substituted at the A- or B-site to enhance the oxygen permeability of perovskite oxides. Anion doping at the oxygen site is also an effective strategy to improve oxygen permeability [11]. Fluoride anion (F^−^) is chosen as a dopant for oxygen due to its high electronegativity. When doped into the perovskite lattice, F^−^ strongly attracts electrons, reducing the valence electron density of oxygen, thereby weakening the chemical bond between the A- or B-site cations and the oxygen ions [12]. This strong attraction facilitates the diffusion of oxygen ions within the perovskite lattice, thereby enhancing the oxygen permeation rate. Previously, our group reported for the first time the doping of F^−^ into the perovskite lattice used for oxygen-permeable membranes, which significantly improved the oxygen permeability and operational stability of the membrane at low temperatures [13]. Moreover, anion substitution has been extensively studied for its impact on enhancing the performance of perovskite oxides. For example, Wang et al. utilized F^−^ and Cl^−^ doping to improve the separation performance of a hydrogen-permeable membrane, achieving a hydrogen permeation flux of 0.2 mL cm^−2^ min^−1^ [14,15]. Additionally, Wang et al. incorporated anions into a perovskite-permeable membrane to improve its stability in CO_2_ environments, applying it in a methane partial oxidation reactor for syngas production, where the oxygen permeation flux reached 5 mL cm^−2^ min^−1^ [16]. Shao et al. reported that F^−^-doped perovskite-type oxide materials at oxygen sites significantly enhanced oxygen reduction reaction performance and improved electron transfer efficiency [17]. These studies demonstrate that anion doping at oxygen sites can substantially improve the performance of perovskite oxides.

A hollow fiber (HF) membrane offers advantages such as a high specific surface area, strong mechanical strength, and scalability in production [18]. However, existing spinning processes present challenges, including a complex procedure, difficulty in precisely controlling the membrane thickness, and susceptibility to defects. Previously, we simplified the production processes of perovskite HF membranes and proposed a one-step thermal processing (OSTP) approach [19]. Different from the traditional production processes, in which perovskite powder is used as the feedstock, the OSTP approach consists of using readily available feedstock chemicals to obtain precursors and then sintering the membranes in a single heating process. The HF membrane precursor must be immersed in an external coagulation bath to complete phase transformation [20]. However, extended soaking may lead to the dissolution of some elements, thereby disrupting the stoichiometric balance, impairing the sintering process, and ultimately resulting in membrane defects [21].

**Figure 1:**
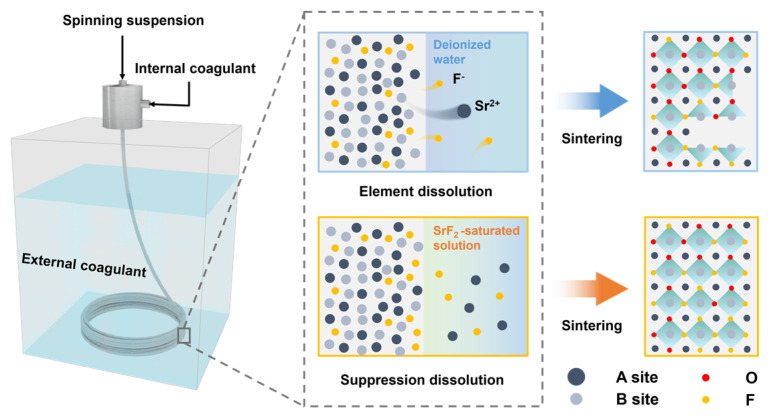
A schematic diagram of BSCFF HF membrane preparation using the traditional OSTP approach and the improved OSTP approach in this work.

Here, we propose a new method for preparing four-channel HF Ba_0.5_Sr_0.5_Co_0.8_Fe_0.2_O_3−δ_F_0.05_ (BSCFF) membranes, which addresses the elemental dissolution issue commonly encountered in traditional OSTP (Figure 1). In traditional OSTP, deionized water is used as the internal and external coagulation baths. However, the elements in BSCFF, particularly F^−^, are soluble in water. As a result, during the preparation of BSCFF membranes using the traditional OSTP approach, F^−^ can easily dissolve in the deionized water, leading to stoichiometric ratio changes. To mitigate elemental dissolution, we replaced deionized water with a saturated solution as the internal and external coagulation baths. Elemental dissolution was effectively prevented, which ensured an accurate stoichiometric ratio of BSCFF membranes. In order to demonstrate the superiority of the improved OSTP approach, we compared the surface chemical state and the solubility in water of BSCF membranes with and without F^−^ doping. Additionally, the sintering behavior, phase stability, lattice structure, and oxygen permeability of the BSCFF membranes were systematically studied. Using this improved OSTP approach, F^−^ was successfully doped into the perovskite lattice, which improved its performance by about 20% compared with a membrane prepared with traditional OSTP.

## 2. Experimental Section

### 2.1. Preparation of HF Membranes

To prepare the BSCFF HF membrane, BaCO_3_, SrCO_3_, Co_2_O_3_, Fe_2_O_3_, and SrF_2_ were ball-milled thoroughly for 24 h in ethanol according to the stoichiometric ratio. The resulting mixture was then dried at 80 °C for 24 h, ground, and sieved through a 300 mesh. The mixed raw chemicals were further mixed with 1-methyl-2-pyrrolidinone (NMP), polyetherimide (PEI), and polyvinylpyrrolidone (PVP) at a mass ratio of 105:40:10:1, and they were ball-milled for 24 h to produce uniform spinning suspensions. The spinning suspensions were subsequently extruded through a tetra-bore spinneret to form HF precursors. The precursors were immersed in an external coagulant for 24 h. Intact HF precursors with asymmetric structures were obtained through the phase inversion process reported previously [19]. The intact HF precursors were sintered at 1120 °C for 5 h in static air after drying to obtain dense HF membranes. The heating and cooling rates were 2 °C min^−1^. All chemicals used had a purity greater than 99.5% and were purchased from Sinopharm Chemical Reagent (Sinopharm, Wuhan, China).

In the traditional OSPT method, deionized water was used as the internal and external coagulation baths in the phase inversion process. The resulting BSCF and BSCFF membranes are denoted as BSCF-H_2_O and BSCFF-H_2_O, respectively. In the improved OSPT method (Figure 1), BSCF HF membranes were prepared by using BSCF-saturated solutions as internal and external coagulation baths, denoted as BSCF-SS. BSCFF HF membranes were prepared by using SrF_2_-saturated solutions as internal and external coagulation baths, denoted as BSCFF-SS. The saturated solution was obtained by adding excess solute to deionized water. The mixture was thoroughly stirred to dissolve the solute in the solution. The mixture was then left to rest until the excess solute precipitated and the solution reached saturation. The fabrication parameters are summarized in Table 1.

### 2.2. Characterizations

The crystal phase structure of the perovskite powders and HF membranes were characterized using X-ray diffraction (XRD, Bruker, model D8 Advance, Billerica, MA, USA) at room temperature (RT), with Cu Kα radiation in the range of 20° ≤ 2θ ≤ 80°. The phase transformation of the mixed raw chemicals for BSCFF membrane preparation from room temperature to 1100 °C and back to room temperature in static air was examined using in situ high-temperature XRD measurements (HT-XRD, Philips, X’ Pert Pro, Eindhoven, The Netherlands). The heating and cooling rates were 10 °C min^−1^. The holding time at each temperature before obtaining the XRD pattern was 0.5 h. The morphology of the HF membranes was observed by using a scanning electron microscope (SEM, Hitachi S-4800, Tokyo, Japan). A thermogravimetry (TG, STA 409 PC Netzsch, Hanau, Germany) analysis of the HF precursors was performed from room temperature to 1100 °C with a heating rate of 10 °C min^−1^ in air. A dilatometer (Netzsch DIL 402 C, Hanau, Germany) was applied to investigate the sintering behavior of the HF precursors in the air at a heating rate of 5 °C min^−1^. Inductively coupled plasma–optical emission spectroscopy (ICP-OES, PE Optima 2000 DV, Perkin Elmer, Waltham, MA, USA) measurement was applied to analyze the element contents in the SrF_2_-saturated solution or deionized water coagulation baths after BSCFF membrane soaking. Supernatants with different soaking days were selected. Each sample was tested at least three times. The particle size distribution of the mixed raw chemicals for BSCFF membrane preparation was measured using Malvern M200 (Malvern, UK) with water as the dispersion medium. The composition of the membrane was verified using energy-dispersive X-ray spectroscopy (EDX, Hitachi S-4800, Tokyo, Japan). X-ray photoelectron spectra (XPS, Thermo ESCALAB 250, Waltham, MA, USA) obtained with an Al Kα X-ray source (hʋ = 1486.6 eV) were utilized to analyze the chemical composition and element valence state of the membranes. The spectra were calibrated via 284.6 eV of C 1s. The mechanical strength of the HF membranes was measured through a three-point bending test, which was performed using a tensile tester (Model CMT6203, MST, Manchester, NH, USA) provided with a load cell of 5 kN. The samples were fixed in the sample holder with a spec gauge length of 20 mm. The crosshead speed was set to 0.02 cm min^−1^. The effective length of the sample was 5 cm. The breaking load (F_m_) needed to break the samples was recorded for each sample.

### 2.3. Oxygen Permeation Measurement

The gas-tightness of the HF membranes was tested using nitrogen at room temperature. The oxygen permeation performances of the membrane were measured using a homemade high-temperature oxygen permeation apparatus, which was reported in our previous study [22]. An HF membrane with a length of 30 was sealed with two quartz tubes. The flow rate of air at the feed side (i.e., the shell side) was 120 mL min^−1^, and the helium flow rate at the sweep side (i.e., the lumen side) was 60 mL min^−1^. The inlet gas flow rates were precisely controlled using mass flow controllers (model D07–19B, Beijing Jianzhong Machine Factory, Beijing, China). The oxygen permeation flux was measured in the temperature range of 550 °C to 900 °C. The effective area was simply defined as the outer surface area because of the complex structure of the HF membrane, and it was approximately 0.85 cm^2^. An online gas chromatograph (Agilent, GC-7820A, Santa Clara, CA, USA) was applied to analyze the composition of the permeate side outlet. The leakage of oxygen due to the imperfect sealing and silver softening at high temperatures was less than 0.5% of the total oxygen permeation flux during all the experiments at elevated temperatures. The long-term stability of the BSCFF-SS membrane was tested with 60 mL min^−1^ He as the sweep gas and 120 mL min^−1^ air as the feed gas at 750 °C. The oxygen concentration of the outlet sweep gas was measured, and the oxygen flux was calculated. To ensure the repeatability of our experimental data, the oxygen permeation measurement of the membrane prepared using each fabrication technique was repeated at least three times. The average was taken as the final result to minimize errors. Supposing that the leakage of N_2_ and O_2_ occurs through pores or cracks according to Knudsen diffusion, the leakage flow rate of N_2_ and O_2_ could be expressed using the following relationship [23]:

(1) $$\frac{J_{N_{2}}^{Leak}}{J_{O_{2}}^{Leak}}={\left(\frac{32}{28}\right)}^{0.5}\times \frac{79}{21}=4.02$$

The oxygen permeation flux was calculated as follows [24]:

(2) $$J_{O_{2}}=(C_{O_{2}}-\frac{C_{O_{2}}}{4.02})\times \frac{Q}{A}$$

Here, $C_{O_{2}}$ and $C_{N_{2}}$ correspond to the $N_{2}$ and $O_{2}$ concentrations measured using GC, Q is the flow rate of the permeate gas stream, and A is defined as the surface effective area of the exposed membrane.

## 3. Results and Discussion

### 3.1. Fabrication of BSCFF HF Membrane

**Figure 2:**
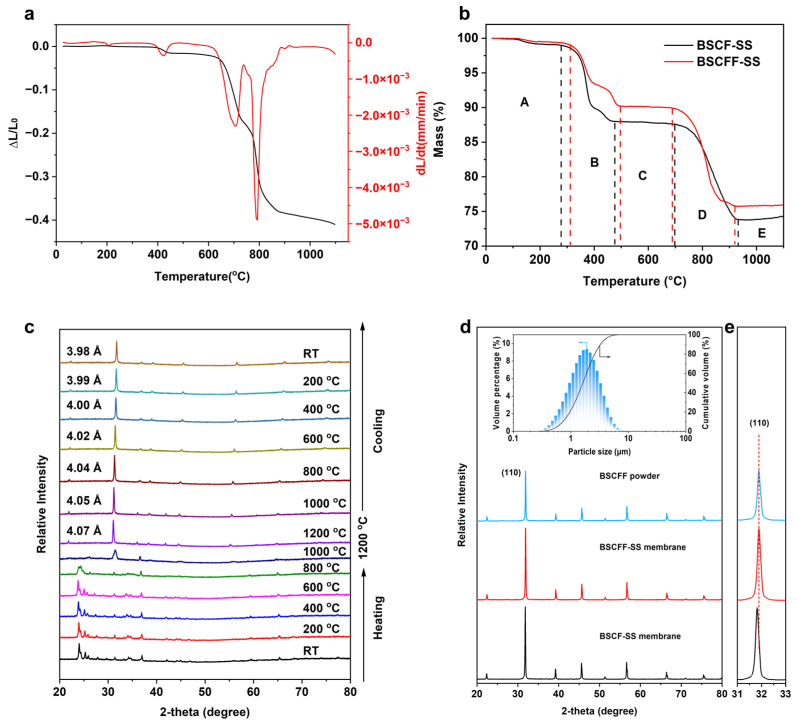
(**a**) The temperature dependence of the thermal expansion ratio and the corresponding derivation curve of the BSCFF-SS HF precursor. (**b**) The temperature dependence of the TG curves of the BSCF-SS and BSCFF-SS HF precursors. (**c**) In situ HT-XRD patterns of the mixed raw chemicals for BSCFF membrane preparation. Inset: particle size distribution of the mixed raw chemicals for BSCFF membrane preparation. XRD pattern (**d**) from 20° to 80° and (**e**) from 31° to 33° of the BSCFF powder, BSCF-SS HF membrane, and BSCFF-SS HF membrane.

The properties of perovskite membranes are greatly affected by sintering conditions, such as temperature, dwell time, atmosphere, and heating and cooling rates. In particular, the sintering temperature is one of the main factors affecting membrane densification. The temperature dependence of the thermal expansion ratio and the corresponding derivation curve of the BSCFF-SS HF precursor are shown in Figure 2a. During the sintering process of the BSCFF-SS precursor at 750–850 °C, the shrinkage rate substantially increased. In this stage, holes created by the combustion of organic matter were gradually filled through element rearrangement, transitioning from a disordered to an ordered state, which resulted in the formation of the perovskite crystal structure and gradual densification [25]. The shrinkage of the BSCFF-SS membrane sintered at 1100 °C reached approximately 41%, indicating a fully densified structure. Typically, a shrinkage rate of around 30% is sufficient to achieve densification in HF membranes [26]. However, for the BSCFF-SS HF precursor, when the shrinkage rate reached 30%, it did not stop shrinking. The sintering plateau appeared at around 1000 °C. The high shrinkage rate of the BSCFF-SS membrane marked the completion of the final densification stage. The thermal decomposition process of the BSCFF-SS and BSCF-SS HF precursors consisted of five distinct stages (Figure 2b). At stage A, the mass of both BSCFF-SS and BSCF-SS decreased slightly due to the volatilization of the residual water and solvent NMP in the precursor [27]. At stage B, the mass of BSCFF-SS decreased by approximately 9%, while BSCF-SS experienced a larger reduction of about 14%. This was primarily attributed to the decomposition of PEI, the organic binder in the precursor. At stage C, the mass of both samples remained relatively stable. At stage D, the mass of the two samples significantly declined by approximately 13%, primarily due to the decomposition of metal carbonates and the in situ formation of composite oxides. At the final stage, stage E, the mass of the two samples remained relatively stable, indicating the formation of the perovskite phase structure and the sintering densification of the HF membranes.

To explore the phase transformation process of BSCFF, in situ HR-XRD of the mixed raw chemicals was performed in static air (Figure 2c). According to the HT-XRD patterns recorded during the heating process (from RT to 1200 °C), the raw material transformed into a perovskite phase at about 1000 °C and remained stable at 1200 °C. During the cooling process (from 1200 °C to RT), the BSCFF oxide retained its original perovskite phase structure without the formation of any impurity phases. A slight shift in the diffraction peaks toward higher angles was observed as the temperature decreased, attributed to the shrinkage of the oxide lattice [28]. The lattice parameter calculated from the HT-XRD patterns decreased from 4.07 Å to 3.98 Å as the temperature decreased. These findings clearly demonstrate that F^−^ doping does not alter the original perovskite structure. The BSCFF perovskite had excellent structural and chemical stability.

The XRD patterns for the BSCFF powder, BSCF-SS, and BSCFF-SS HF membranes showed similar perovskite structures with no detectable impurities (Figure 2d). Additionally, the particle size distribution of the mixed raw chemicals is also a key factor affecting the sintering behavior of HF precursors and, ultimately, membrane morphology and performance [29]. The mixed raw chemicals exhibited a concentrated particle size distribution, with an average particle size of 1.86 μm, free from agglomerates (Figure 2d inset). This promoted uniform oxide particle dispersion in the spinning solution, effectively minimizing the defects in the resulting HF membranes. In the magnified view of the primary diffraction peaks, it was observed that F^−^ doping in the BSCF oxides caused a shift in the diffraction peaks toward higher diffraction angles, indicating a reduction in the lattice parameter and lattice shrinkage of the perovskite [30] (Figure 2e). Inserted F^−^ can replace the oxide ions (O^2−^) in the perovskite lattice, causing lattice shrinkage, or it can be introduced into the interstitial position, causing lattice expansion [31]. F^−^ doping led to the lattice shrinkage of BSCFF, indicating that F^−^ was successfully doped into the perovskite lattice.

**Figure 3:**
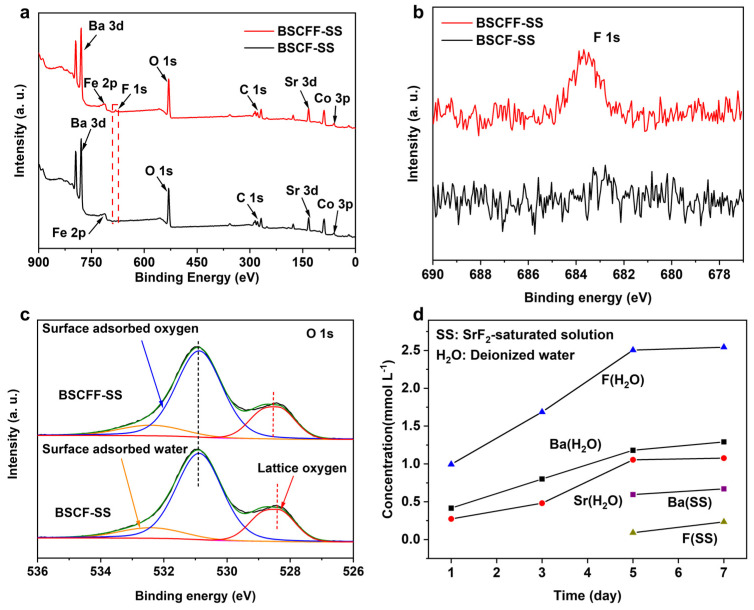
(**a**) XPS full survey spectra, (**b**) F 1s spectra, and (**c**) O 1s spectra of BSCF-SS and BSCFF-SS. (**d**) Element contents in SrF2-saturated solution or deionized water coagulation baths after BSCFF membrane soaking, obtained via ICP-OES measurements.

The presence of the F element in BSCFF-SS was further confirmed by XPS spectra (Figure 3a). The peaks observed at binding energies of approximately 779 eV, 133 eV, 61 eV, 712 eV, 531 eV, 684 eV, and 268 eV corresponded to Ba 3d, Sr 3d, Co 3p, Fe 2p, O 1s, F 1s, and C 1s spectra, respectively. No F 1s XPS peaks were identified in the BSCF-SS membrane, confirming the successful incorporation of F into the BSCF membrane, consistent with the XRD results (Figure 3b). The actual doping amount of F^−^ was calculated through quantitative analysis using EDX and XPS (Table 2). The calculated doping amounts and target doping amounts of F^−^ were similar. The proportion of F^−^ indicates that it was effectively doped into the membrane. Furthermore, we also investigated the influence of F^−^ doping on the metal–oxygen bond energy. The O1s XPS spectrum consisted of three peaks located at 528.5 eV, 530.9 eV, and 532.4 eV, corresponding to lattice oxygen, oxygen species adsorbed on the surface, and water molecules adsorbed on the surface, respectively (Figure 3c) [32]. The binding energy of the lattice oxygen in BSCFF-SS shifted slightly toward a higher value than that of BSCF-SS [33]. The increased binding energy of the lattice oxygen in BSCFF-SS could be attributed to the electronegativity of F^−^ (3.98) being stronger than that of O^2−^ at 3.44. Thus, F^−^ attracts more valence electrons from the metal ions than oxygen ions, resulting in a lower valence electron density of O^2−^. This phenomenon weakens the metal–oxygen bonds and consequently increases the mobility of lattice oxygen and protons [13].

The difference in the elemental dissolution of the BSCFF-H_2_O HF precursors in the deionized water and SrF_2_-saturated solution coagulation baths demonstrates the superiority of the improved OSTP approach (Figure 3d). During the first 5 days, no dissolved elements were detected in the SrF_2_-saturated solution. After this point, only minimal increases in the F and Ba concentrations were observed. In contrast, in deionized water, the concentration of F reached 1 mmol L^−1^ within just 1 day, with the dissolution of Ba and Sr also being detected. The elements’ content dissolved in deionized water increased over time. After 5 days, elemental dissolution began to slow down. The dissolved F after 7 days amounted to 66.7% of the total doped F. The phase conversion process can be fully completed within 5 days, so the saturated solution can replace deionized water as the internal and external coagulation baths in OSTP, effectively inhibiting element loss and accurately controlling the stoichiometric ratio of the HF membranes.

**Figure 4:**
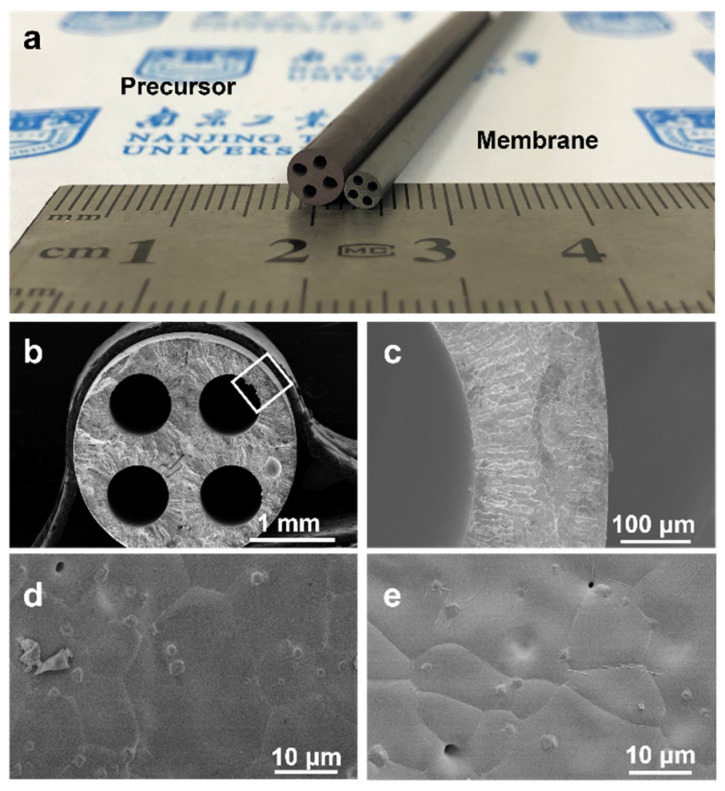
(**a**) BSCFF-SS HF precursor (**left**) and membrane (**right**). SEM images of BSCFF-SS HF membrane: (**b**) cross-section overview, (**c**) in region of skeleton marked on (**b**,**d**) inner surface, (**e**) outermost surface.

A photograph of the BSCFF-SS HF precursor and the sintered HF membrane shows that they had well-formed four-channel asymmetric structures (Figure 4a). The SEM images of the BSCFF-SS HF membrane revealed that its outer and channel diameters were approximately 2.54 mm and 0.72 mm, respectively (Figure 4b–e). The four-channel asymmetric structure featured dense layers on both the inner and outer surfaces. Finger-like pores extended inward from the inner and outer surfaces, converging at a middle dense layer. The presence of these finger-like pores contributed to a relative reduction in mass transfer resistance. Although some small pores remained on the inner and outer surfaces after sintering, they did not compromise the gas-tightness of the membrane, as they were not interconnected.

Mechanical strength is a critical factor in determining the service life of HFs. A three-point bending strength test was conducted on the BSCFF-SS HF membranes obtained at different sintering temperatures. The corresponding F_m_ values are presented in Table 3. The F_m_ of the membranes sintered at temperatures higher than 1050 °C was greater than 30 N, indicating good mechanical strength and exceeding the F_m_ of the BSCF membranes reported in previous studies [34]. However, it was only when the sintering temperature reached 1120 °C that the membrane surface became dense and thus met the gas-tightness requirements of oxygen separation. The corresponding F_m_ was about 39.62 N. The high mechanical strength of the BSCFF-SS membranes enhances their potential for commercial applications in oxygen separation membranes.

### 3.2. Oxygen Permeation

**Figure 5:**
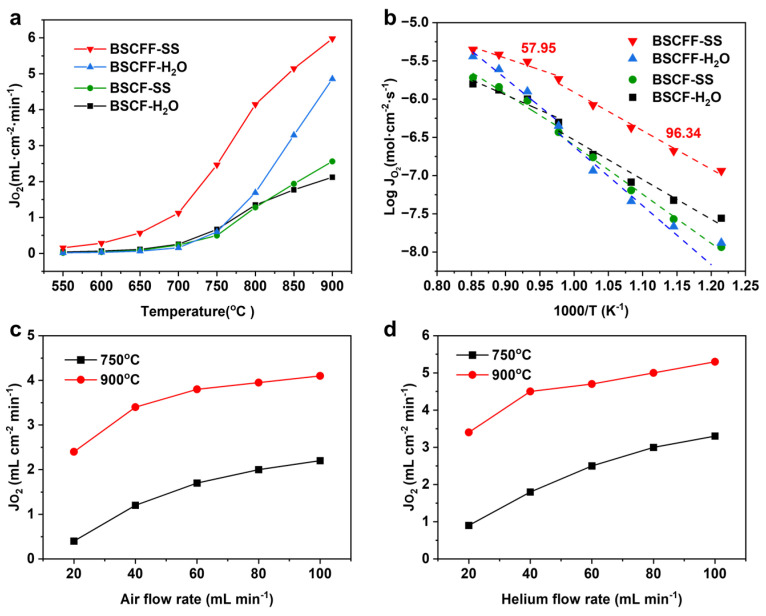
(**a**) Temperature dependence on the oxygen permeation flux; (**b**) Arrhenius plots of BSCFF and BSCF HF membranes in different coagulation baths. (**c**,**d**) Sweep gas flow rate dependence on the oxygen permeation flux of the BSCFF-SS membrane at 750 °C and 900 °C.

An evaluation of oxygen permeability offers valuable insights into the effects of the improved OSTP approach on the performance of HF membranes. Different fabrication techniques can significantly influence the microstructure of membranes and, consequently, their oxygen transport properties. Figure 5a depicts the temperature dependence of oxygen permeation flux. The permeation measurement of the membranes prepared using each fabrication technique was conducted at least three times to ensure repeatability. The oxygen permeation fluxes of the membranes were in the following order: BSCFF-SS > BSCFF-H_2_O > BSCF-SS > BSCF-H_2_O. When the temperature was 700 °C, the oxygen permeation flux of the BSCFF-SS membrane was already greater than 1 mL cm^−2^ min^−1^. This value meets the minimum requirements for commercial applications of oxygen separation membranes [35]. BSCFF-SS had the highest oxygen permeation flux (6.2 mL cm^−2^ min^−1^) at 900 °C, which was approximately 21% higher than that of BSCFF-H_2_O (4.9 mL cm^−2^ min^−1^) and three times higher than that of BSCF-SS (2.5 mL cm^−2^ min^−1^). Regarding BSCF, the oxygen permeation flux of BSCF-SS prepared using the improved OSTP approach was also 19% higher than that of BSCF-H_2_O (2.1 mL cm^−2^ min^−1^) at 900 °C. This suggests that F^−^ doping and utilizing a saturated solution as the internal and external coagulation bath can effectively prevent elemental dissolution and enhance the oxygen permeation flux.

A nonlinear relationship with two activation energies (E_a_) was observed for the BSCF and BSCFF membranes in the Arrhenius plots (Figure 5b). The turning point occurred at 750 °C, attributed to the order–disorder transition of the oxygen vacancies [36]. At this temperature, perovskites undergo crystal structure changes, such as a transition from cubic to hexagonal. The E_a_ of BSCFF-SS was 57.95 kJ mol^−1^ between 900 °C and 750 °C, and it was 96.34 kJ mol^−1^ between 750 °C and 550 °C. BSCFF-SS exhibited the lowest activation energy, indicating reduced mass transfer resistance, which facilitated oxygen permeation. The oxygen permeation of BSCF and BSCFF membranes is governed by both bulk diffusion and surface exchange reactions. When the temperatures are below 700 °C, the oxygen permeation of the membrane is mainly controlled by the surface exchange reactions. Therefore, the oxygen flux of the BSCFF-H_2_O membrane was not significantly different from that of the BSCF-SS and BSCF-H_2_O membranes. As the bulk diffusion and the surface exchange increased along with the rising temperature, the oxygen flux increased significantly. The oxygen permeability of the BSCFF-H_2_O membrane was relatively inferior to that of the BSCFF-SS membrane due to element dissolution. The oxygen permeation flux was influenced by the differences in the final stoichiometric ratios resulting from the use of different coagulation baths.

Elevating the flow rates of the feed gas and sweep gas can increase the chemical potential gradient across the membrane and thus increase the oxygen flux. We compared the effects of air (feed gas) (Figure 5c) and helium (sweep gas) flow rates (Figure 5d) on the oxygen permeation flux of the BSCFF-SS membrane at 750 °C and 900 °C. The oxygen permeation flux for both increased with the flow rate, with the increase in the helium flow rate having a greater impact. When the temperature was greater than 750 °C and the flow rate was greater than 20 mL min^−1^, the oxygen permeability of the membranes all exceeded 1 mL cm^−2^ min^−1^, which also meets the requirements for commercial applications of oxygen separation membranes.

### 3.3. Long-Term Stability

**Figure 6:**
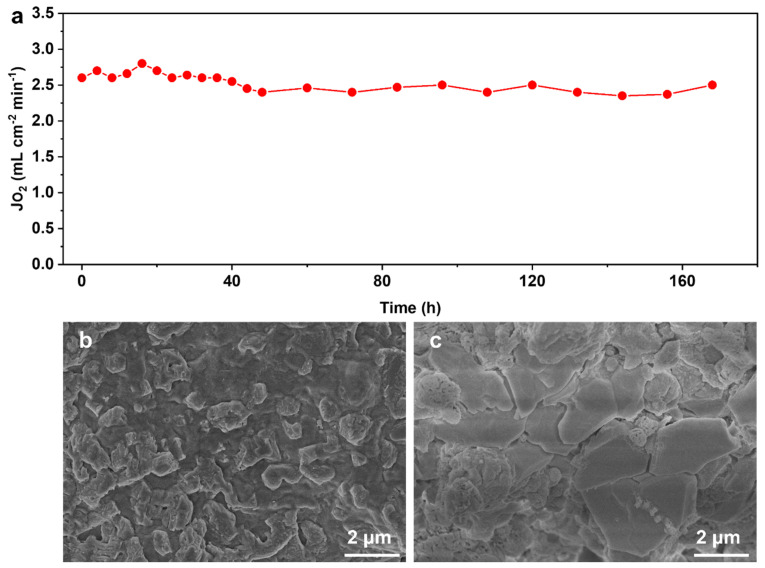
(**a**) Long-term stability of BSCFF-SS HF membrane (temperature = 750 °C; F_Air_ = 120 mL·cm^−2^·min^−1^; F_He_ = 60 mL·cm^−2^·min^−1^). SEM images of the BSCFF-SS HF membrane after long-term operation: (**b**) inner surface, (**c**) outermost surface.

Long-term operational stability is a crucial aspect of the performance of oxygen separation membranes, particularly in practical applications. We conducted a stability test on the BSCFF-SS HF membrane (Figure 6a). During the 168 h continuous test, the oxygen flux was maintained at approximately 2.5 mL cm^−2^ min^−1^ without significant attenuation, consistent with the oxygen permeation measurement results in Figure 5a. The surface morphologies of the air and sweep sides of the BSCFF-SS membrane after the long-term stability test were characterized using SEM (Figure 6b,c). The inner and outermost surfaces of the spent BSCFF-SS membrane had different morphologies, which could be attributed to the effect of oxygen partial pressure on element segregation. At high temperatures and under an oxygen partial pressure gradient, the cations in the membrane tend to segregate, leading to an enrichment of A- and B-site elements on the sweep and feed sides [37]. This structural alteration often impacts oxygen permeability, explaining why most perovskite membranes exhibit a gradual decline in oxygen flux during prolonged operation. However, after 168 h of testing, the inner and outermost surface morphologies of the BSCFF-SS HF membrane were almost unchanged. There was also no decrease in the oxygen permeation flux. Although the grain boundary on both the feed and the sweep sides was partially corroded and became unclear, the BSCFF-SS membrane was still dense without through holes. These results demonstrate the reliability and stability of the BSCFF-SS membrane in long-term operation.

Although the BSCFF-SS HF membrane exhibits excellent oxygen permeability and long-term stability, there are still some challenges. The performance of the membrane may be affected under extreme environmental conditions. The production costs and scalability required for membrane scale-up need to be further optimized. Nevertheless, the BSCFF-SS HF membrane is very promising for energy, environmental, and industrial gas separation.

## 4. Conclusions

In summary, using saturated solutions instead of deionized water as the internal and external coagulation baths during membrane preparation effectively suppresses elemental dissolution. Compared with deionized water, the use of a saturated solution as the coagulation bath increases the oxygen flux of the membrane by 20%. This approach ensures that F^−^ is successfully doped according to the target stoichiometry, facilitating the establishment of new oxygen ion transport pathways in the membrane based on the ability of F^−^ to weaken the chemical bonds between A/B-site cations and oxygen ions. This reduces the activation energy for oxygen permeation, significantly enhancing oxygen flux. At a low temperature of 700 °C, the oxygen flux of the BSCFF-SS membrane reaches the commercial applications of 1 mL min^−1^ cm^−2^, substantially outperforming the more established BSCF membranes. The introduction of F^−^ expands the potential of perovskite oxides for oxygen permeation, particularly in low-temperature applications. The preparation method proposed in this work optimizes the fabrication process of perovskite HF membranes for industrial production, enhancing product quality and offering applicability in fields such as material manufacturing.

## Figures and Tables

**Table 1:** The fabrication parameters of the HF membranes.

Fabrication Parameters	Value
Spinning temperature	20 °C
Injection rate of internal coagulant	10 mL min^−1^
Injection rate of suspension	4 mL min^−1^
Air gap	0 cm

**Table 2:** The atomic ratios of the elements in the BSCFF-SS HF membrane determined using EDX and XPS analyses.

Element	EDX/Atomic%	XPS/Atomic%	Target/Atomic%
Ba	13.69	13.97	10
Sr	8.05	8.47	10
Co	11.05	7.07	16
Fe	3.72	2.22	4
O	62.27	67.05	59
F	1.21	1.23	1

**Table 3:** The F_m_ and gas-tightness of the BSCFF-SS HF membranes obtained at different sintering temperatures.

Temperature (°C)	Character (mm)	F_m_ (N)	Gas-Tightness
1050	OD = 2.76 ID = 0.75	37.12 ± 0.83	Gas leaking
1060	OD = 3.24 ID = 0.75	45.25 ± 1.08	
1070	OD = 2.79 ID = 0.75	39.56 ± 2.53	
1080	OD = 3.15 ID = 0.75	38.37 ± 0.73	
1090	OD = 3.24 ID = 0.75	39.65 ± 1.61	
1100	OD = 3.09 ID = 0.75	45.97 ± 1.30	
1110	OD = 3.03 ID = 0.75	38.39 ± 0.96	
1120	OD = 2.97 ID = 0.75	39.62 ± 1.28	Gas-tight

## Data Availability

The original contributions presented in this study are included in the article. Further inquiries can be directed to the corresponding authors.
